# Leucine-enriched essential amino acids attenuate inflammation in rat muscle and enhance muscle repair after eccentric contraction

**DOI:** 10.1007/s00726-016-2240-1

**Published:** 2016-05-11

**Authors:** Hiroyuki Kato, Kyoko Miura, Sayako Nakano, Katsuya Suzuki, Makoto Bannai, Yoshiko Inoue

**Affiliations:** Frontier Research Laboratories, Institute for Innovation, Ajinomoto Co., Inc., Kawasaki, Kanagawa Japan

**Keywords:** Eccentric contractions, Muscle repair, Leucine-enriched essential amino acids, Mammalian target of rapamycin, Inflammation, Interleukin-6

## Abstract

Eccentric exercise results in prolonged muscle damage that may lead to muscle dysfunction. Although inflammation is essential to recover from muscle damage, excessive inflammation may also induce secondary damage, and should thus be suppressed. In this study, we investigated the effect of leucine-enriched essential amino acids on muscle inflammation and recovery after eccentric contraction. These amino acids are known to stimulate muscle protein synthesis via mammalian target of rapamycin (mTOR), which, is also considered to alleviate inflammation. Five sets of 10 eccentric contractions were induced by electrical stimulation in the tibialis anterior muscle of male SpragueDawley rats (8–9 weeks old) under anesthesia. Animals received a 1 g/kg dose of a mixture containing 40 % leucine and 60 % other essential amino acids or distilled water once a day throughout the experiment. Muscle dysfunction was assessed based on isometric dorsiflexion torque, while inflammation was evaluated by histochemistry. Gene expression of inflammatory cytokines and myogenic regulatory factors was also measured. We found that leucine-enriched essential amino acids restored full muscle function within 14 days, at which point rats treated with distilled water had not fully recovered. Indeed, muscle function was stronger 3 days after eccentric contraction in rats treated with amino acids than in those treated with distilled water. The amino acid mix also alleviated expression of interleukin-6 and impeded infiltration of inflammatory cells into muscle, but did not suppress expression of myogenic regulatory factors. These results suggest that leucine-enriched amino acids accelerate recovery from muscle damage by preventing excessive inflammation.

## Introduction

Eccentric contractions occur when a force applied to muscle exceeds the momentary force generated by the muscle itself (Lindstedt et al. 2002). Such contractions impact the human body in both adverse and favorable ways, as higher maximum force can be generated than during concentric or isometric contractions. Consequently, training that incorporates eccentric exercise results in greater gains in muscle strength and size (Higbie et al. 1996). On the other hand, eccentric contractions are also well known to induce muscle damage and dysfunction lasting from several days to several weeks, and thereby reduce the ability to perform athletic activities and potentially prevent regular exercise (Cleak and Eston 1992). Therefore, strategies to mitigate adverse effects may promote general health and athletic performance.

Initial muscle damage induced by mechanical stress triggers prominent local inflammation to remove cellular debris (Tidball 1995). In particular, increased Ca^2+^ influx due to disruption of the cell membrane in mechanically stressed skeletal muscle induces expression of the pro-inflammatory cytokine interleukin-6 (IL-6) (Juretic et al. 2006). In turn, IL-6 recruits and activates inflammatory cells to produce a host of cytotoxic substances, including superoxide anions and hydrogen peroxide (Brickson et al. 2001; Liu and Spolarics 2003; Nguyen and Tidball 2003; Tidball 1995). By producing additional pro-inflammatory cytokines (Cannon and St Pierre 1998), infiltrating inflammatory cells may induce secondary muscle damage. On the other hand, IL-6 is also essential for regeneration, a process controlled by the myogenic regulatory factors MyoD, Myf5, myogenin, and MRF4. For instance, IL-6 regulates myoblast proliferation and differentiation via MyoD and myogenin (Serrano et al. 2008), and controls macrophage migration during muscle regeneration (Zhang et al. 2013). Therefore, to improve muscle repair, it is important to suppress the pro-inflammatory effects of IL-6 without inhibiting regenerative activity.

Leucine has well known to stimulate the mammalian target of rapamycin (mTOR), also known as the potent stimulator of protein synthesis (Crozier et al. 2005). Indeed, recent reports demonstrated that mTOR is a key pathway in inflammation-dependent muscle regeneration, and that mTOR overexpression attenuates inflammation in cardiomyocytes, and prevents cardiac dysfunction (Song et al. 2010). Also, the potent mTOR inhibitor rapamycin impairs muscle regeneration after injury (Ge et al. 2009). Accordingly, some reports have shown that administration of amino acids (Nosaka et al. 2006), particularly leucine (Kirby et al. 2012) or branched-chain amino acids (BCAAs) (Jackman et al. 2010), suppresses delayed-onset muscle soreness and blood creatine kinase activity, which are typical symptoms of muscle damage. However, due to some limitations of the parameters measured (Clarkson and Newham 1995), the impact of amino acids on recovery from muscle damage was not definitively established. Indeed, the most reliable markers of muscle damage are histology and muscle function (Paulsen et al. 2012). However, repeated biopsies may also elicit increased inflammation in muscle (Malm 2001). Also, a small muscle biopsy might not be sufficiently representative of the whole muscle (Beaton et al. 2002). Thus, it is necessary to analyze the whole muscle to understand the recovery process.

In neonatal pigs (Escobar et al. 2005) and humans (Borgenvik et al. 2012), administration of leucine or a mixture of BCAAs elicits a decrease in plasma concentrations of other essential amino acids. However, essential amino acids other than leucine are also required to sustain leucine-induced synthesis of muscle protein (Kobayashi et al. 2006). Indeed, leucine-enriched essential amino acids can stimulate muscle protein synthesis via mammalian target of rapamycin (mTOR) (Drummond and Rasmussen 2008). Therefore, we hypothesized that leucine-enriched amino acids may enhance muscle repair by modulating inflammation. Thus, we investigated the effects of leucine-enriched essential amino acids on muscle damage, inflammatory response, and expression of myogenic factors after eccentric contractions in rats.

## Materials and methods

### Animals

Male SpragueDawley rats 8–9 weeks old (Charles River Laboratories Japan, Inc., Yokohama, Japan) were housed in a temperature-controlled room on a 12-h light–dark cycle, and provided water and CR-F1 standard commercial chow (Charles River Laboratories Japan, Inc., Yokohama, Japan) ad libitum.

### Experimental design

**Fig. 1:**
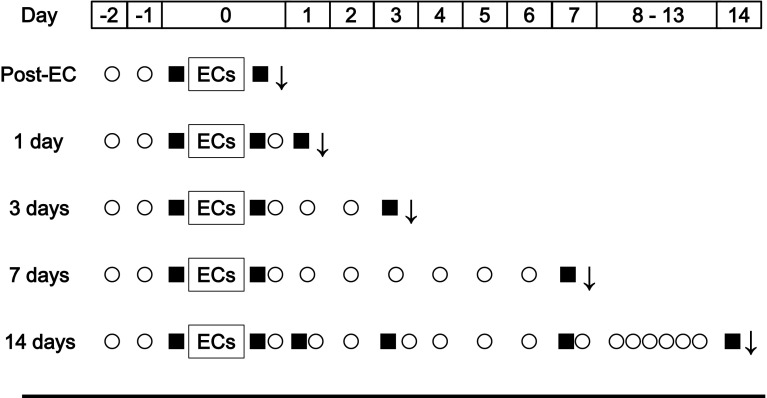
Overall experimental design. Rats treated with distilled water (EC-Con, *n* = 24) and leucine-rich essential amino acids (EC-AminoL40, *n* = 25) underwent electrical stimulation under anesthesia to induce five sets of 10 eccentric contractions. Sedentary rats (*n* = 24) treated with distilled water were used as control. Muscle function was evaluated by measuring maximum isometric dorsiflexion under electrical stimulation. The tibialis anterior muscle was excised immediately after eccentric contraction (Post-EC, *n* = 4), and 1 (*n* = 5), 3 (*n* = 5), 7 (*n* = 5), and 14 days (*n* = 5–6) thereafter. *Downwards arrow* tissue sampling; *filled square* measurement of muscle function; *circle* daily dosing with leucine-enriched essential amino acids or water

The study design is illustrated in Fig. 1. Sedentary rats received distilled water by oral gavage throughout the experiment (Sed, *n* = 24). Rats similarly treated with distilled water underwent electrical stimulation to induce eccentric contraction (EC-Con, *n* = 24). Finally, a group of rats that also underwent electrical stimulation to induce eccentric contraction received oral doses of leucine-enriched essential amino acids instead (EC-AminoL40, *n* = 25). It was previously confirmed that daily administration of amino acids did not affect food intake. Under inhalation anesthesia with 1.5 % isoflurane, rats were killed immediately (*n* = 4), 1, 3, 7 days (*n* = 5), and 14 days (*n* = 5–6) after eccentric contraction. The tibialis anterior muscle was collected at various time points. The mid-belly of the muscle was fixed by neutralized 10 % formalin for histochemistry. The remaining tissue was frozen in liquid nitrogen, and stored at −80 °C until gene expression analysis. The muscle samples collected 14 days after eccentric contraction were not used for these analyses, because, muscle damage and gene expression recovered within 7 days after eccentric contraction.

### Eccentric contraction

Eccentric contraction was induced as previously described (Mori et al. 2014). Briefly, animals were fasted for 3 h, and electrically stimulated to induce a total of five sets of 10 eccentric contractions, each set separated by 1 min. The tibialis anterior muscle was stimulated percutaneously under inhalation anesthesia with 1.5 % isoflurane, using a pair of surface electrodes. Electrical stimuli were applied for 1100 ms with constant current 4–5 mA, frequency 100 Hz, and pulse duration 1 ms via a SEN-3301 electrical stimulator (Nihon Kohden Corp., Tokyo, Japan) fitted with an SS-202J isolator (Nihon Kohden Corp., Tokyo, Japan). The muscle was simultaneously stretched over 900 ms from an ankle position of 45° to 135° using a customized NDH-1 device (Bio Research Center Co., Ltd., Nagoya, Japan), beginning at 200 ms after the start of electrical stimulation.

### Leucine-enriched essential amino acids

Leucine-enriched amino acids (1 g/kg body weight) were administered by oral gavage once a day, beginning at 2 days prior to eccentric contraction until killing. On the day of eccentric contraction, amino acids were administered immediately after the rats came out of anesthesia, a process that took 30 min. The mixture consisted of 2 % histidine, 11 % isoleucine, 40 % leucine, 17 % lysine, 3 % methionine, 7 % phenylalanine, 9 % threonine, 1 % tryptophan, and 11 % valine (Ajinomoto Co., Inc., Tokyo, Japan). The composition is based on the ratio of essential amino acids in whey except that leucine is enriched, and was developed specifically to prevent decreased availability of other essential amino acids due to a high dose of leucine (Bukhari et al. 2015). All control animals received distilled water by oral gavage once a day over the same period.

### Muscle function

Under inhalation anesthesia with 1.5 % isoflurane, maximum isometric dorsiflexion torque was measured prior to (Pre-EC; *n* = 24–25) and immediately after eccentric contraction (Post-EC; *n* = 24–25), as well as 1, 3, 7 (*n* = 10–11), and 14 days after stimulation (*n* = 5–6). Maximal dorsiflexion was elicited using supramaximal tetanic current with train duration 650 ms, pulse 1 ms at 100 Hz, constant current 4–5 mA, and both knee and ankle joints set at 90°.

### Histochemical analysis

The tibialis anterior muscle was aligned in cross-section, and immersed in neutralized 10 % formalin for at least 3 days, and embedded in paraffin. Subsequently, cross-sections (5 µm thick) from paraffin-embedded tissue were stained with hematoxylin and eosin. Muscle fibers with nuclei stained by hematoxylin and infiltrated with inflammatory cells were considered damaged, and damage was assessed by point counting (Chang et al. 2003). Briefly, four regions were randomly selected in each cross-section, and covered with a 792-point grid at 20× magnification to mark points over damaged muscle fibers. Damage is reported as percentage of damaged grid points. Histochemistry was assessed by investigators who were blinded to experimental conditions.

### Semi-quantitative real-time PCR

Gene expression of cytokines and myogenic regulator factors was determined using semi-quantitative real-time PCR. Total RNA was extracted using RNeasy Fibrous Tissue Mini Kit (QIAGEN, Valencia, CA, USA). Yield was measured using Nanodrop 1000 (Thermo Scientific, Waltham, MA, USA), and quality was assessed by the ratio of absorbance at 260–280 nm. Total RNA (1 µg) was then reverse transcribed using PrimeScript RT Master Mix (Takara, Ohtsu, Japan). RNA and cDNA samples were then stored at −80 °C until further analysis. Relative mRNA expression was determined by real-time PCR using TP800 Thermal Cycler Dice Real-Time System (Takara Bio, Ohtsu, Japan) and SYBR Premix Ex Taq Tli RNaseH Plus (Takara Bio, Ohtsu, Japan). The details concerning these primers are shown in Table 1. Glyceraldehyde-3-phosphate dehydrogenase was used as an internal control, and relative fold change was determined from cycle threshold (*C*_T_) values using the 2^−∆∆CT^ method (Livak and Schmittgen 2001).

**Table 1:** Primers for real-time quantitative PCR. GAPDH, glyceraldehyde-3-phosphate dehydrogenase; IL-6, interleukin-6; IL-1β, Interleukin-1β

Gene	Accession no.	Forward primer	Reverse primer
GAPDH	NM_017008	GGCACAGTCAAGGCTGAGAATG	ATGGTGGTGAAGACGCCAGTA
Myogenine	NM_017115	CCAGTGAATGCAACTCCCACA	GTAGGGTCAGCTGCGAGCAA
MyoD	NM_176079	CACACCTCTGACAGGACAGGACA	TTCTGACGGTTGGAATGCACA
IL-6	NM_001008725	ATTGTATGAACAGCGATGATGCAC	CCAGGTAGAAACGGAACTCCAGA
IL-10	NM_031512	CTACCTATGTCTTGCCCGTGGAG	GGGAACATCACACACTAGCAGGTC
MuRF-1	AY059627	TGACCAAGGAAAACAGCCACCAG	TCACTCCTTCTTCTCGTCCAGGATGG

### Measurement of amino acid concentrations in blood and muscle

Plasma samples were deproteinized by precipitation with 5 % sulfosalicylic acid (1:1) and centrifugation for 10 min at 10,000 rpm and 4 °C. On the other hand, TA muscle was pulverized using Multi-beads shocker (Yasui Kikai Corporation, Osaka, Japan), extracted with 17 volumes of 5 % sulfosalicylic acid, and centrifuged for 10 min at 10,000 rpm and 4 °C. The supernatant was filtered at 10,000 rpm and 4 °C for 30 min using Amicon Ultra 0.5 mL (Merck Millipore, Billerica, MA, USA). Amino acid concentrations in plasma and muscle were measured on an automatic amino acid analyzer (JLC-500; JEOL, Tokyo, Japan).

### Statistical analysis

Values are reported as mean ± SEM. All variables were examined by two-way ANOVA, with treatment and time as factors. When a significant main effect or interaction was observed, Bonferroni’s multiple comparisons test was used to compare groups. Data were analyzed in GraphPad Prism 5 (GraphPad Software Inc., San Diego, CA, USA), with *p* < 0.05 considered significant.

## Results

### Body weight and muscle weight

**Fig. 2:**
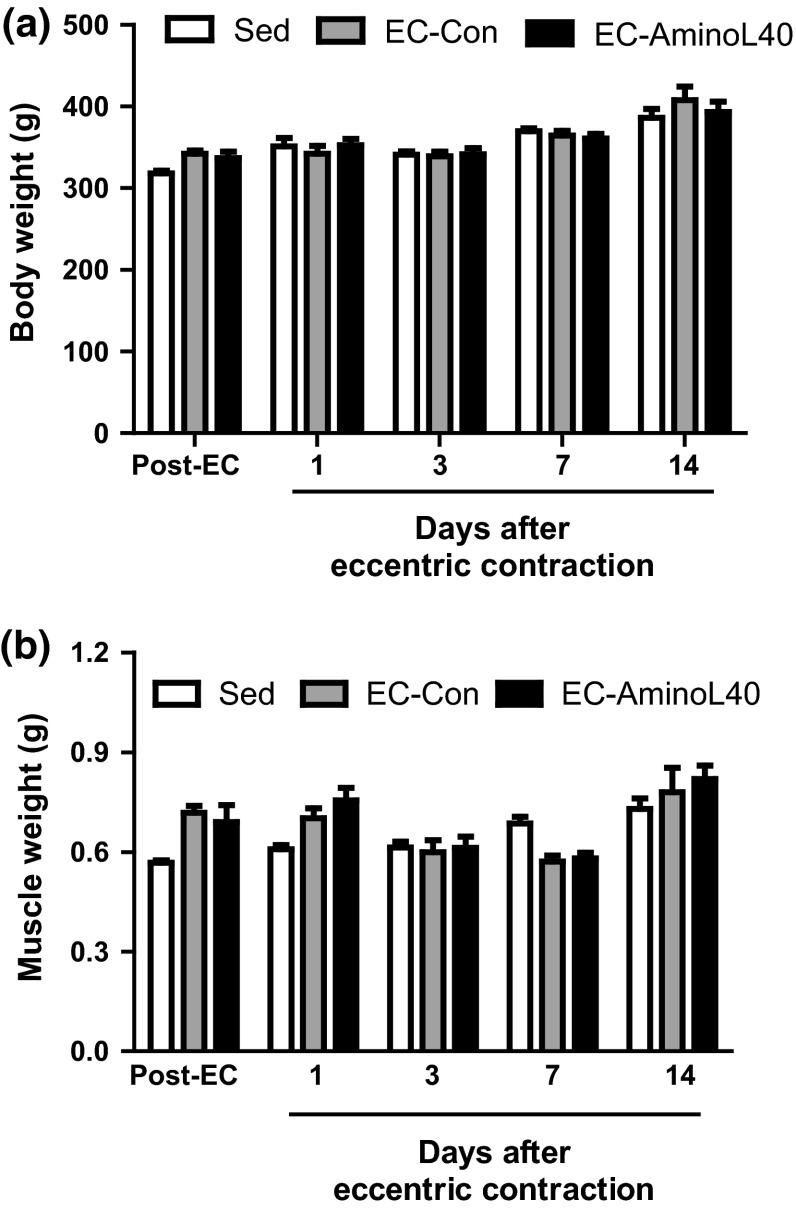
Body weight (**a**) and weight of the tibialis anterior muscle (**b**) immediately after (Post-EC), and 1, 3, 7, and 14 days after eccentric contraction. Data are mean ± SEM (**a** *n* = 5–25, **b** *n* = 4–6). There was no significant difference at all time points among sedentary rats treated orally with water (Sed), and rats that underwent eccentric contractions and were treated orally with water (EC-Con) or leucine-enriched essential amino acids (EC-AminoL40)

There was no significant difference in body weight and the weight of the tibialis anterior muscle among groups at all time points (Fig. 2).

### Muscle function

**Fig. 3:**
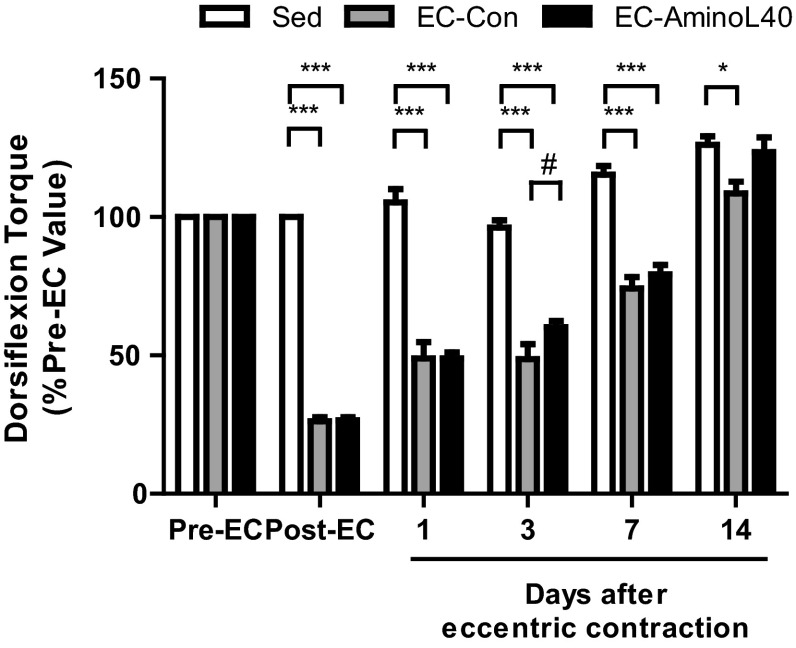
Time course of maximum isometric dorsiflexion torque. Maximum isometric dorsiflexion torque was measured before (Pre-EC, *n* = 24–25), immediately after (Post-EC, *n* = 24–25), as well as 1, 3, 7 (*n* = 10–11), and 14 days (*n* = 5–6) after eccentric contraction. Data are mean ± SEM. * and ^#^, *p* < 0.05; ***, *p* < 0.001

Maximum isometric dorsiflexion torque gradually increased over 14 days in Sed rats (Fig. 3) as a result of muscle growth. Maximum isometric dorsiflexion torque significantly decreased immediately after eccentric contraction (Post-EC) relative to sedentary rats (*p* < 0.001), but gradually increased over the next 14 days. Indeed, there were significant differences throughout the experiment between sedentary rats and rats that underwent eccentric contraction. Notably, rats that received leucine-enriched essential amino acids (EC-AminoL40) gained within 14 days the same level of isometric dorsiflexion torque as in Sed rats, while rats dosed with distilled water (EC-Con) presented lower isometric dorsiflexion torque than Sed rats, indicating incomplete recovery. Also, rats treated with amino acids regained stronger muscle function within 3 days relative to rats treated with distilled water (*p* < 0.05).

### Histochemical features

**Fig. 4:**
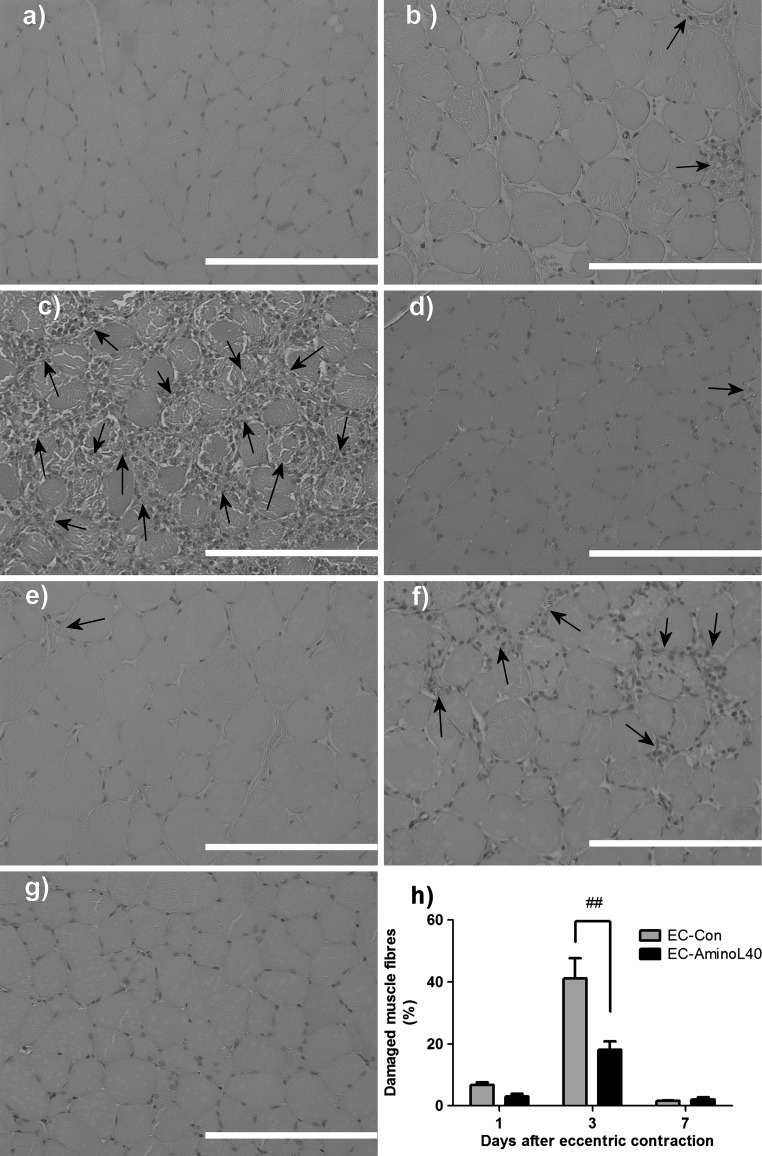
Histochemical analysis of the tibialis anterior muscle before and after eccentric contraction. Hematoxylin and eosin staining of transverse sections of the muscle in **a** sedentary animals (Sed), and from **b**–**d** rats that underwent eccentric contraction and received water (EC-Con) or **e**–**g** leucine-enriched essential amino acids (EC-AminoL40). Tissues were collected **b**, **e** 1 day, **c**, **f** 3 days, and **d**, **g** 7 days after eccentric contraction. Images are representative sections from 4–5 rats killed at a given time point, with magnification ×40. *Scale bars* 100 μm. **h** Muscle damage 1, 3, and 7 days after eccentric contraction, as assessed by point counting, and expressed as *percentage* of muscle fibers infiltrated with inflammatory cells (*arrows*) in each sampling grid. Data are mean ± SEM (*n* = 4–6). ^##^, *p* < 0.001

Inspection of sections stained with hematoxylin and eosin did not show morphological changes in muscle fibers or presence of inflammatory cells in sedentary rats (Fig. 4a). In contrast, damaged muscle fibers were observed 1, 3, and 7 days after eccentric contraction (Fig. 4b–g). In rats treated with distilled water, damage due to eccentric contraction peaked at 41 % of the total area of the tibialis anterior muscle within 3 days (Fig. 4h). Notably, the number of damaged muscle fibers was significantly lower in rats treated with leucine-enriched amino acids than in animals treated with distilled water (*p* < 0.001, Fig. 4h).

### Gene expression

**Fig. 5:**
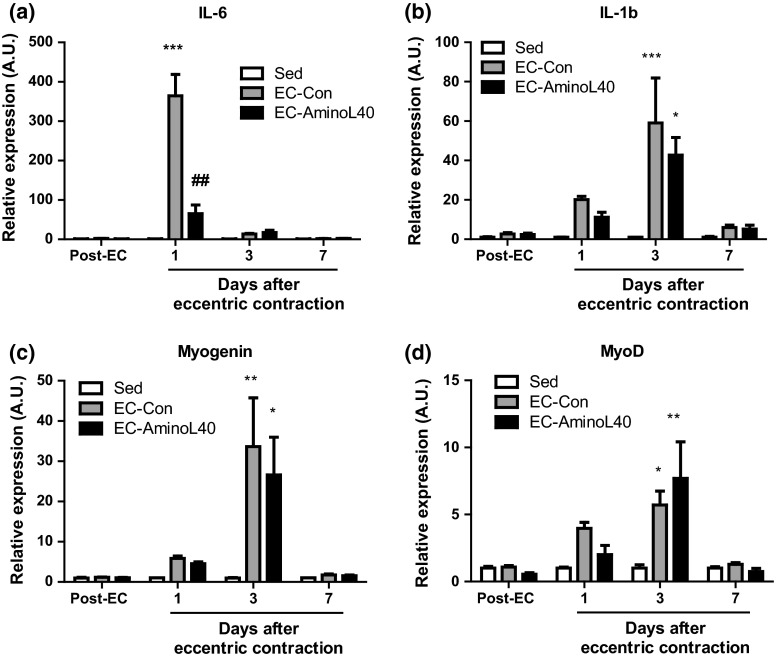
Time course of gene expression in muscle tissue. The tibialis anterior muscle was obtained at various time points from sedentary rats (Sed), and from rats that underwent eccentric contraction and treated with distilled water (EC-Con) or leucine-enriched essential amino acids (EC-AminoL40). Samples were analyzed by real-time PCR to quantify mRNA transcripts of IL-6 (**a**), IL-1β (**b**), myogenin (**c**), and MyoD (**d**). Gene expression was normalized to glyceraldehyde-3-phosphate dehydrogenase, and is reported as fold-increase relative to sedentary rats at each time point. Data are mean ± SEM (*n* = 4–6). *, **, and ***, *p* < 0.05, 0.01, 0.001 vs. sedentary rats; ^##^, *p* < 0.01 between EC-Con and EC-AminoL40 rats

Expression of all genes tested did not change immediately after eccentric contraction (Fig. 5). However, IL-6 expression significantly increased 1 day after eccentric contraction in rats treated with distilled water (*p* < 0.001 vs. sedentary rats). Subsequently, IL-6 levels returned to baseline within 3–7 days (Fig.5a). Leucine-rich essential amino acids significantly suppressed IL-6 expression 1 day after eccentric contraction (*p* < 0.001). Expression of IL-1β, myogenin, and MyoD increased within 3 days after eccentric contraction, but to levels comparable between rats treated with distilled water and those treated with leucine-enriched amino acids (Fig. 5b–d).

### Amino acid concentrations

The concentrations of essential amino acids in plasma and muscle tissue are listed in Tables 2 and 3. Plasma Met decreased 1 day after eccentric contraction. Following LEAA administration, plasma His decreased in comparison with Sed rats, while plasma Leu and Met decreased relative to EC-Con rats. On the other hand, muscle His and Thr increased the day following eccentric contraction, and Ile, Leu, Met, Phe, and Val rose 2 days thereafter. However, LEAA administration significantly decreased His concentration 3 days after eccentric contraction.

**Table 2:** Plasma amino acid concentrations (μM) were measured 2 h after measurement of muscle function

	Group	Post-EC	1 day	3 days	7 days
His	Sed	69 ± 4	66 ± 1	67 ± 1	67 ± 1
	EC-Con	69 ± 2	61 ± 1	60 ± 1	60 ± 2
	EC-AminoL40	78 ± 1	56 ± 2*	68 ± 0	65 ± 3
Ile	Sed	93 ± 7	100 ± 8	91 ± 1	92 ± 2
	EC-Con	93 ± 6	88 ± 3	83 ± 2	84 ± 3
	EC-AminoL40	108 ± 6	88 ± 4	101 ± 3	100 ± 2
Leu	Sed	163 ± 10	177 ± 14	163 ± 4	165 ± 4
	EC-Con	168 ± 14	158 ± 6	149 ± 4	148 ± 4
	EC-AminoL40	194 ± 11	156 ± 6	183 ± 6^+^	177 ± 4
Lys	Sed	355 ± 15	337 ± 37	402 ± 17	389 ± 11
	EC-Con	336 ± 10	324 ± 17	374 ± 13	362 ± 12
	EC-AminoL40	348 ± 14	322 ± 22	406 ± 10	380 ± 14
Met	Sed	85 ± 0	83 ± 3	79 ± 4	67 ± 2
	EC-Con	78 ± 4	63 ± 4**	67 ± 3	73 ± 1
	EC-AminoL40	89 ± 3	76 ± 3	83 ± 5^+^	77 ± 4
Phe	Sed	71 ± 4	75 ± 2	76 ± 1	75 ± 4
	EC-Con	64 ± 2	70 ± 4	70 ± 1	68 ± 1
	EC-AminoL40	70 ± 1	67 ± 3	76 ± 1	75 ± 1
Thr	Sed	290 ± 19	292 ± 17	317 ± 13	313 ± 10
	EC-Con	344 ± 24	292 ± 18	297 ± 11	298 ± 5
	EC-AminoL40	383 ± 26	305 ± 40	326 ± 25	293 ± 9
Trp	Sed	97 ± 7	102 ± 4	83 ± 6	109 ± 9
	EC-Con	79 ± 5	90 ± 4	84 ± 8	93 ± 3
	EC-AminoL40	105 ± 6	88 ± 5	105 ± 5	94 ± 5
Val	Sed	206 ± 16	225 ± 17	211 ± 4	208 ± 6
	EC-Con	221 ± 17	196 ± 7	193 ± 6	190 ± 6
	EC-AminoL40	254 ± 12	201 ± 12	234 ± 9	223 ± 5

Values are mean ± SE (*n* = 4–5)

*^,^** *P* < 0.05, 0.01 significantly different from the Sed group

^+^ *P* < 0.05 significantly different from the EC-Con group

**Table 3:** Intramuscular amino acid concentrations (μM) were measured 2 h after measurement of muscle function

	Group	Post-EC	1 day	3 days	7 days
His	Sed	266 ± 19	270 ± 14	328 ± 5	306 ± 19
	EC-Con	283 ± 16	396 ± 44**	267 ± 16	251 ± 22
	EC-AminoL40	247 ± 20	328 ± 26	167 ± 15**^,+^	187 ± 30
Ile	Sed	131 ± 9	125 ± 9	129 ± 5	121 ± 5
	EC-Con	143 ± 6	152 ± 12	205 ± 21**	115 ± 6
	EC-AminoL40	134 ± 13	147 ± 7	199 ± 14**	110 ± 5
Leu	Sed	176 ± 12	179 ± 12	177 ± 9	164 ± 7
	EC-Con	198 ± 8	230 ± 17	338 ± 35**	171 ± 8
	EC-AminoL40	194 ± 14	226 ± 11	318 ± 23**	176 ± 5
Lys	Sed	1260 ± 177	1191 ± 140	1565 ± 65	1429 ± 69
	EC-Con	1158 ± 120	1164 ± 87	1204 ± 100	1875 ± 150
	EC-AminoL40	965 ± 99	1129 ± 129	1076 ± 48**	1766 ± 148
Met	Sed	85 ± 7	80 ± 2	63 ± 8	69 ± 6
	EC-Con	104 ± 9	114 ± 7	146 ± 22**	3 ± 11
	EC-AminoL40	99 ± 6	109 ± 8	148 ± 11**	77 ± 5
Phe	Sed	96 ± 3	91 ± 3	98 ± 7	89 ± 4
	EC-Con	93 ± 3	116 ± 11	154 ± 10**	87 ± 4
	EC-AminoL40	89 ± 6	112 ± 5	137 ± 5**	89 ± 6
Thr	Sed	855 ± 49	818 ± 30	989 ± 35	859 ± 33
	EC-Con	908 ± 82	1332 ± 96**	1246 ± 41	1163 ± 50
	EC-AminoL40	876 ± 105	1360 ± 206**	144 ± 85	913 ± 38
Trp	Sed	N.D.	N.D.	N.D.	N.D.
	EC-Con	N.D.	N.D.	N.D.	N.D.
	EC-AminoL40	N.D.	N.D.	N.D.	N.D.
Val	Sed	259 ± 21	255 ± 13	256 ± 11	236 ± 8
	EC-Con	287 ± 18	318 ± 23	376 ± 29**	232 ± 10
	EC-AminoL40	270 ± 18	304 ± 18	363 ± 18**	232 ± 3

Values are mean ± SE (*n* = 4–5)

*ND* not detected

** *P* < 0.01 significantly different from the Sed group

^+^ *P* < 0.05 significantly different from the EC-Con group

## Discussion

We found that leucine-rich essential amino acids attenuated IL-6 expression in mechanically stressed muscles, in line with data obtained during recovery from endurance exercise (Rowlands et al. 2016). However, further studies based on immunohistochemistry and protein expression of cytokines and myogenic factors are required to elucidate the molecular mechanisms underlying this effect. One possibility is that the amino acids modulate inflammation and IL-6 expression via mTOR. Indeed, mTOR overexpression suppresses IL-6 secretion from cardiomyocytes exposed to lipopolysaccharides (Song et al. 2010), as well as inflammation in the heart after ischemia–reperfusion injury (Aoyagi et al. 2012). Also, mTOR activation reduces activation and expression of intercellular adhesion molecule 1 in endothelial cells, and thereby inhibits neutrophil invasion (Minhajuddin et al. 2009). Collectively, these observations indicate that mTOR may alleviate inflammation in some tissues and organs. Also, further studies using our model are also required to investigate acute changes in the inflammatory response due to administration of leucine-enriched amino acids, especially in light of a previous study, in which administration after endurance exercise transiently activated a pro-inflammatory network centered on IL-1β within 30 min, although inflammatory and myogenic activity decreased by 240 min (Rowlands et al. 2016).

Alleviation of excessive inflammation is traditionally believed to enhance muscle repair (Urso and Sawka 2013). Thus, the apparent ability of leucine-enriched amino acids to mitigate muscle damage likely depends on its ability to attenuate excessive inflammation due to IL-6, which is expressed in inflammatory and skeletal muscle cells (Paulsen et al. 2012), and believed to amplify the inflammatory response by inducing monocyte differentiation into macrophages and to increase the oxidative burst (Kaplanski et al. 2003). We note that IL-6 may also be involved in degrading muscle proteins during pathologic muscle wasting (Strassmann et al. 1992; Tsujinaka et al. 1995). However, IL-6 is also essential for muscle regeneration (Zhang et al. 2013), because, it regulates myoblast proliferation and differentiation (Serrano et al. 2008). Thus, whether anti-inflammatory agents should be taken to enhance recovery from exercise remains an open question (Urso and Sawka 2013).

Accordingly, we found that leucine-enriched essential amino acids also alleviated muscle dysfunction, which is correlated with the extent of muscle damage (McCully and Faulkner 1986; Mori et al. 2014). Notably, these amino acids restored full muscle function 14 days after eccentric contraction without an observable increase in muscle mass, in line with previous results demonstrating that leucine accelerated connective tissue repair and functional recovery after cryolesion muscle damage (Pereira et al. 2014b), and also reduced macrophage infiltration into muscle tissue (Pereira et al. 2014a). One possibility is that leucine-enriched amino acids help prevent inflammation-induced fibrosis (Abdelmagid et al. 2012), which may interfere with tissue repair and functional recovery (Stauber 2004). Nevertheless, further studies are warranted to investigate whether these amino acids should be used to minimize muscle fibrosis after exercise.

Due to lack of isonitrogenous control, we cannot exclude the possibility that the beneficial effects of leucine-enriched essential amino acids may be due to leucine alone, especially in light of reports indicating that branched-chain amino acids like leucine decrease markers of muscle damage in rats (Pereira et al. 2014a, b) and humans (Jackman et al. 2010; Kirby et al. 2012; Nosaka et al. 2006; Shimomura et al. 2010), including creatine phosphokinase and muscle soreness. In addition, branched-chain amino acids also accelerated recovery of maximum voluntary contraction after squat exercises (Shimomura et al. 2010). However, the impact on muscle function after exercise remains controversial, as amino acids were found in some studies to have no effect on muscle recovery (Jackman et al. 2010; Nosaka et al. 2006). Leucine was also found to decrease peak force (Kirby et al. 2012). Thus, further studies comparing isonitrogenous controls and different mixtures of amino acids or proteins may help identify the optimal composition of amino acids. However, even if this were the case, the identity of such amino acid may no longer be needed in practical terms, especially since other essential amino acids rather than leucine or branched-chain amino acids are needed to maintain the increase in muscle protein synthesis induced by leucine (Kobayashi et al. 2006). Also, LEAAs have been investigated to stimulate muscle protein synthesis after several types of exercise (Dreyer et al. 2008; Pasiakos et al. 2011). Thus, the mixture of leucine-enriched amino acids, rather than branched-chain amino acids or leucine alone, may have sufficient biological activity in the end to promote post-exercise recovery by alleviating muscle damage, enhancing muscle adaptation, and increasing protein synthesis.

Following oral administration, amino acid concentrations transiently increase, and return to basal levels within 9 h (Lynch et al. 2006). Thus, plasma amino acids were comparable between EC-Con and EC-AminoL40 rats, because, amino acids were not administered on the morning of the sampling day. Notably, most plasma amino acids were not affected by eccentric contraction, but higher intracellular concentrations of Ile, Leu, Met, Phe, and Val were observed 3 days later, which may indicate increased protein breakdown. On the other hand, LEAA administration did not alter the concentration of essential amino acids except Lys and His, implying that exogenous LEAAs probably do not affect the degradation of muscle protein.

In summary, we found that daily administration of leucine-enriched amino acids restored full muscle function 14 days after eccentric contraction, an effect not achieved using a similar regimen of distilled water. Indeed, muscle function was stronger within 3 days in rats treated with amino acids than in those treated with distilled water. We also found that leucine-enriched amino acids modulated IL-6 expression 1 day after contraction, as well as the ensuing muscle damage 3 days after contraction. However, the amino acids did not impact expression of the myogenic regulatory factors MyoD and myogenin. Taken together, the results suggest that the amino acids enhance muscle repair by suppressing excessive inflammation without impeding regeneration.

## Abbreviations

IL-6: Interleukin-6
LEAAs: Leucine-enriched essential amino acids
mTOR: Mammalian target of rapamycin
